# Surgical leadership within rapidly changing working conditions in Germany

**DOI:** 10.1515/iss-2019-0002

**Published:** 2019-04-22

**Authors:** Thomas Schmitz-Rixen, Reinhart T. Grundmann

**Affiliations:** Department of Vascular and Endovascular Surgery, Goethe-University-Hospital, Theodor-Stern-Kai 7, 60590 Frankfurt/Main, Germany; German Institute for Vascular Public Health Research, Berlin, In den Grüben 144, 84489 Burghausen, Germany

**Keywords:** distress, economy, gender, Generation Y, surgery, working conditions, work-life balance

## Abstract

**Introduction:**

An overview of the requirements for the head of a surgical department in Germany should be given.

**Materials and methods:**

A retrospective literature research on surgical professional policy publications of the last 10 years in Germany was conducted.

**Results:**

Surveys show that commercial influences on medical decisions in German hospitals have today become an everyday, predominantly negative, actuality. Nevertheless, in one survey, 82.9% of surgical chief physicians reported being very satisfied with their profession, compared with 61.5% of senior physicians and only 43.4% of hospital specialists. Here, the chief physician is challenged. Only 70% of those surveyed stated that they could rely on their direct superiors when difficulties arose at work, and only 34.1% regarded feedback on the quality of their work as sufficient. The high distress rate in surgery (58.2% for all respondents) has led to a lack in desirability and is reflected in a shortage of qualified applicants for resident positions. In various position papers, surgical residents (only 35% describe their working conditions as good) demand improved working conditions. Chief physicians are being asked to facilitate a suitable work-life balance with regular working hours and a corporate culture with participative management and collegial cooperation. Appreciation of employee performance must also be expressed. An essential factor contributing to dissatisfaction is that residents fill a large part of their daily working hours with non-physician tasks. In surveys, 70% of respondents stated that they spend up to ≥3 h a day on documentation and secretarial work.

**Discussion:**

The chief physician is expected to relieve his medical staff by employing non-physician assistants to take care of non-physician tasks. Transparent and clearly structured training to achieve specialist status is essential. It has been shown that a balanced work-life balance can be achieved for surgeons. Family and career can be reconciled in appropriately organized departments by making use of part-time and shift models that exclude 24-h shifts and making working hours more flexible.

## Introduction

Chief physicians are increasingly facing economic constraints, a shortage of junior staff, an increasing proportion of women in medicine, and demands from junior staff for a balanced work-life balance. This means that economic expertise and leadership competence in personnel matters, in addition to the core competence as medical decision maker and surgeon, are increasingly in demand from chief physicians. Employee motivation has become another core competence. Some aspects – especially concerning the situation in Germany – are discussed in the following sections.

## Commercialization of patient-related decision making in hospitals

A qualitative study [1] with the premise that a hospital must generate a profit to secure its existence has been conducted by Wehkamp and Naegler. The increase in the number of cases and the case mix indices in Germany suggest that admission, treatment, and discharge of patients are not only influenced by medical aspects but are also profit oriented, i.e. they have become commercialized. In a pilot study, 31 managing directors and 32 physicians were asked to what extent they were observing this development in their everyday professional life. From the physicians’ point of view, economic influences on medical decisions in German hospitals are now an almost daily and predominantly negative actuality. Not one interviewee positively assessed commercialization or welcomed it. Surveyed managers repeatedly emphasized that they had no desire to admit patients without medical indications for inpatient treatment. However, physicians reported that, in addition to indirect influence, direct influence on medical decisions was also being exercised or attempted. From a medical perspective, the strongest indirect influences include staffing assessment and management decisions about opening or closing departments and wards. In many hospitals, the purchasing department makes decisions according to cost and not according to aspects of medical quality. The majority of physicians regard the executive management group as the central power in hospitals, making decisions with regard to recruitment, dismissal, staffing, corporate strategy and objectives, bonus payments, key points of resource allocation, and the closure or opening of wards. The chain of command moves from the management level via the chief physicians, who are generally obligated to follow economically relevant target agreements, down to the senior physicians.

For most physicians, the personnel policy of management is a provocative issue. Residents say they are not receiving sufficient specialist background training. They skid into extreme situations of overburdening, where they must make decisions alone, under time pressure, and without sufficient expertise – untenable in view of their responsibility toward patients and the hospital (see below, “Burnout and distress”). The chief surgeon is asked to counter this development as far as possible, not only for the benefit of entrusted patients, but also to provide a solid specialist training in surgery and prevent health problems in the staff.

In Missives of the Professional Association of German Surgeons (*Mitteilungen des Berufsverbands der Deutschen Chirurgen*), Wienke [2] has, from a legal point of view, commented on the possibilities and limits of economic influences on medical-therapy decisions. He points out that most chief physicians commit themselves to economic aspects in their decision making when they sign the clause in Section 3 of the Chief Physician Model Contract of the German Hospital Society. There, it states: “The physician is obligated to treat patients in the context of the medically necessary with purposeful and economic handling within the available means of the hospital.” Chief physicians must therefore guarantee compliance with current medical standards and are at the same time subject to economic efficiency, appropriateness, necessity, and expediency of the medical service. In practice, the choice of therapy repeatedly gives rise to conflict and a Damocles’ sword hovers over the chief physician. On the one hand, freedom of medical treatment must be guaranteed. On the other hand, the hospital operator requires economic efficiency and has the right to issue instructions under labor law. In the event of a discrepancy, physicians are to refer to their medical competency and seek an open dialogue with the hospital operator and commercial management, as well as with the applicable health insurance. If, during such a discussion, the commercial management or employer is nevertheless prompted to enforce economically influenced measures, doctors should again present their medical motives in writing. In this way, they can prove that they have tried to treat their patients according to standard. However, Wienke also states: “The number of those who courageously oppose the orders of the hospital owners and carry out a more cost-intensive measure will, for understandable reasons, remain low in the future.”

## Leadership with objectives

Vogd et al. [3] sociologically analyzed leadership with objectives (Table 1) and described the chief physician’s situation. In monthly meetings with chief physicians, figures collected by medical controlling – broken down by department – are published and discussed in plenary sessions in many clinics. The number and severity of patient cases are among the most common figures, and serve as benchmarks to be compared with earlier figures or with those of colleagues. Certain numbers thus correspond to performance and are the subject for ad hoc evaluation by colleagues and superiors. Interpreting the evaluation, however, is not cut and dry, as even constant case numbers can be evaluated as either stagnation or solid continuity. “Leadership with objectives” represents a “leadership standard,” within the framework of which a target state is defined and a difference to the actual state can be identified. Awareness of how one is viewed by management and which criteria are being applied to assess performance is important. Every chief physician should know how many patients are being treated and whether the medical objectives (set by management!) are being achieved. Objectives (primarily numbers) are formulated, “handed to the physicians,” or, more unobtrusively formulated, “made transparent.” Every leadership requires the led to agree to be led, but here it is not only a question of letting oneself be led, but of self-leadership, namely based on the objectives.

**Table 1:** Objectives of business management guidelines for the surgical director (according to Wehkamp and Naegler [1]).

–	Annual results
–	Profit
–	Return
–	Contribution margin
–	Number of patients treated in hospital
–	Number of diagnostic and therapeutic services, case mix points
–	Revenue
–	Number of employees
–	(Personnel) costs
–	Case mix points/physician
–	Specifications of the flat rate per case catalogue regarding stay duration

A further aspect of the concept “leadership with objectives” is the so-called bonus or variable portion of the chief physician’s salary. A certain portion of the salary depends on performance. To make such remuneration possible in the first place, certain decisions are requisite. One function the chief physician is expected to carry out is to influence the number of patients admitted to the hospital. If case numbers are no longer bonus-worthy, then implementing employee appraisals is. Here, one must prove that a training plan for the department is in place, that overtime has been reduced a certain percentage in that year, or that vacation time has been reduced (and so on).

## Burnout and distress

In a survey conducted by the German Society of Surgery, 40% of surgeons considered their quality of life worse than that of the general population and 32% even considered it worse than that of their patients [4]. In another survey conducted by Klein et al. [5] in 489 German hospitals (response rate 65%), 48.7% of 1311 surgical hospital physicians fulfilled self-assessed burnout criteria. In the same survey, about a quarter of the respondents suffered from an occupational gratification crisis. A fifth had thought of giving up their profession several times a month or more, and 44% of the surveyed physicians saw the quality of patient care, sometimes or often, impaired by their overwork [6]. Nearly 80% were too exhausted by their work load to address personal interests.

Is it work time or work load that leads to a high burnout rate? In an analysis of 435 German continuing education residents in various specialist disciplines, a total of 17% reported a high level of occupational stress and 9% had a high level of depressive symptoms [7]. Of all the disciplines, physicians in surgery and internal medicine had the highest rate of occupational stress. Nevertheless, among all disciplines, surgical residents in training had the highest ability level and the lowest burnout rate (the highest rate of depression was observed among anesthesiologists). Not so much the time at work, but rather work load and dissatisfaction with the working environment contributed to a high burnout rate. Wegner et al. compared the work time and work load of Hamburg hospital physicians in 1997 and 2007 [8], [9]. Under the title “Fewer hours, more work” [8], they observed a higher burnout risk in 2007 than 10 years previously, despite an average weekly work-time reduction of about 5 h. Of those questioned, 31.4% were at risk of burnout in 2007. According to the authors, further shortening of working hours therefore does not lower high burnout rates. This statement is identical to that of others who have denied a clear relationship between work time and psychological well being [10]. Other factors, such as colleague interaction and the relationship with superiors, were of greater importance in reducing burnout.

Bauer and Groneberg have described the high stress potential of surgery with regard to working conditions [11]. In their study, they evaluated a total of 1142 questionnaires from hospital physicians in the field of surgery. In this study, 58.2% of the respondents reported unfavorable working conditions, resulting in stress. At the same time, about half of the respondents (52.2%) were very satisfied with their profession. Among the respondents who experienced unfavorable working conditions, only 32.5% were still professionally very satisfied. Those experiencing favorable working conditions were much more satisfied (91.4%). Generally, chief physicians had the lowest distress prevalence (22.0%). The highest distress prevalence (66.7%) was seen among specialists. A look at occupational satisfaction showed that the specialists were significantly less satisfied with their occupation than those in other functional positions (43.4%). Senior physicians (61.5%) and chief physicians (82.9%) were more satisfied. The authors accounted for differing distress prevalence with differing scopes of action, and pointed out that improving surgical working conditions could help maintain personnel potential. As specialists had the highest distress prevalence, this was where intervention was most necessary. The authors suggested that the relationship between professional requirements and scope of action (personal commitment and recompense) be restored to a healthy equilibrium. The chief surgical physician is called upon to facilitate this. Only 70% of those surveyed said that they could rely on their direct superior when difficulties at work arose, and only 34.1% regarded feedback on the quality of their work as sufficient. Accordingly, of all medical fields analyzed by Bernburg et al. [7], surgical department heads landed at the bottom of the list when assessed by residents in training in regard to feedback at work and leadership quality.

## Shortage of new surgeons

Vallböhmer et al. [12] recently published a survey of German surgical clinics, titled “Do we now take anyone?” The result was sobering. A general shortage of candidates was seen by 80% of respondents and a shortage of qualified applicants was seen by 94%. The recruiting standards for prospective residents in training have been lowered, and 55% of respondents stated that the majority of their current applicants do not have a German university degree. Almost 80% of applicants had not completed a doctoral thesis. This situation was seen as having a negative impact on the quality of care by 88% of the respondents. When asked how to react to the current shortage of applicants, the surveyed participants felt that it is primarily the hospital’s duty but that it is also the duty of professional and specialist associations to make surgery more attractive. In particular, working conditions, work time models, and further training regulations were seen to need improvement. The majority rejected hiring more surgical technical assistants in response to the applicant shortage. However, comments were also made, for example that the surgery department would benefit from appreciative interaction between all colleagues and flattened hierarchies. Students are often deterred by the autocratic and hierarchical behavior of chief and senior physicians, who themselves have been otherwise trained. Positive reinforcement is often lacking. “No remonstrance is praise enough” prevails.

## What does the younger generation want?

In a literature survey, Ganschow [13] asked why the career aspiration “general surgeon” has steadily declined among medical students in recent years and reaches a low point after the practical year. Above all, the desire for a balanced relationship between work and leisure discourages many students from a surgical career. Long working hours and little free time contrast with the desired lifestyle of many graduates. As a starting point to overcome a threatening shortage in residents, Ganschow viewed making general surgery more attractive for women. Conditions should be adapted to create a more family-friendly environment. This means that while adapting conditions, the surgical director must still fulfill the demand for further training. However, not only transparent continued education contributes to making general surgery more attractive. The central result of the studies was that positive role models and positive internship experience can steer students toward a surgical career. It has been shown in various countries that the percentage of students interested in surgery can be increased both through voluntary and compulsory internships. Internships, clinical traineeships, and the internship year offer the opportunity to set up individual mentoring programs tailored to the needs of the respective educational level. Such programs could, in the future, counteract the considerable loss of interest in a surgical career during the course of medical school.

Based on a survey of 52 ongoing doctors in their practical year (Arzt im Praktikum), mentoring programs for students who are willing to begin further training as surgeons, or at least consider surgical training, has also been recommended by Chiapponi et al. [14]. Surveyed students had completed parts of their surgical internship in two clinics and were asked how they felt about the stress at work. They were also asked to assess the stress experienced by the residents in training. It turned out that the students’ stress was not significantly different from that of the residents. Nevertheless, students rated the residents’ stress as much higher than their own (and higher than that seen by all the other physicians in the department). All students believed that the surgical residents are more stressed by uncertainty than the trained surgeons. In conclusion, despite a stay of 4 months in a surgical clinic, the students were not realistically viewing surgical training and had overestimated its burden. To attract new talent to surgery, it is therefore urgently necessary to give students a better insight into the daily life of a surgeon, with an emphasis on resources and opportunities to reduce professional stress.

Kasch et al. [15] asked 9079 medical students 21 questions about future job choice and what they expected regarding job satisfaction in a nationwide online survey. Most of these students are the future physicians in German hospitals and belong to Generation Y. They expect work-life balance, career opportunities, professional standards, a good working atmosphere, and prestige. For a future medical profession, work-life balance was the most important factor for job satisfaction, with regulated working hours taking first place. Second place was career, comprising aspects of income, career opportunities, scientific work, and a positive attitude toward the performance principle. A high willingness to perform is therefore a characteristic of Generation Y and does not necessarily contradict the desire for regular working hours. A further expectation concerns professional demands. Here, it is not so much about a classical career but rather of general conditions, such as the quality of everyday work, the desire for independent and varied medical activities, good interdisciplinary cooperation, and opportunities for further education and training. Kasch et al. [15] also emphasized that the potential for action of chief physicians becomes particularly clear when the working atmosphere is considered. A corporate culture with a participative management style and collegial cooperation with qualified employees is as much expected as the appreciation of one’s own performance. This insight coincides with studies repeatedly verifying hierarchy/management style or the contact tone as satisfaction guarantees, especially for the young in medical professions.

The “Young Surgeons” Perspective Forum of the German Society of Surgery has also commented on work-life balance [16]. In addition to the points already mentioned by Kasch et al. [15], it was pointed out that just <35% of the surveyed residents would rate their working conditions as good or very good. Young residents spend a large part of their daily working time on non-physician activities. That contributes to major dissatisfaction, leaving them with the impression that they are “more secretary than doctor.” They consider this a “waste of the resource: medical working time.” Surveys show that 70% of colleagues spend up to ≥3 h a day on documentation and secretarial work, and that no comprehensive relief is being provided.

Essential to increasing the satisfaction of young colleagues, without changing the working hours, is a transparent and fair planning system for operations, which guarantees objective, catalogue-conform further training. The authors [16] write: “as a young surgeon you want to operate, that’s why we choose the profession.” Departments offering good surgical training should not be afraid of a transparent electronic surgical catalogue. It increases the satisfaction of their own employees by objectivity and at the same time provides a competitive advantage on the applicant market. It must be made clear that short-term hospital administration policy incorporating an austerity program to curtail further training carries the risk of a future junior staff shortage. In the long run, patient care by well-trained personnel can then not be guaranteed by such hospitals.

The Surgical Working Group of Young Surgeons of the German Society for General and Visceral Surgery has also examined these questions in a position paper: “What do young surgeons want?” What can be done to keep residents and young specialists in surgery? How can the fascination of surgery be conveyed in such a way that medical students choose further training courses to become a general or visceral surgeon [17]? The paper, with the subtitle “Modern Requirements for Surgical Heads,” is addressed directly to the heads of surgical departments. In this paper, particular emphasis is placed on further training for general and visceral surgeons. It says: “The most important building block and central core on the way to becoming a general or visceral surgeon is a structured and transparent continuing education.” Ideally, this should be understood as part of corporate strategy. It includes a fixed continuing education curriculum with a clearly defined rotation plan to enable early and concrete planning of each individual’s continued training. Training components should include regular further training discussions and feedback, assistance with partial operational steps, release for further training measures, practical internal clinical practice options such as suture courses, SkillsLab or laparoscopy training, keeping a logbook, and mentoring programs. Assistance with partial steps within the framework of various interventions has proven to be particularly important in learning operative skills and procedures. To permanently establish such a concept, the surgical chief physician must serve as a role model by exemplifying assistance for partial steps.

When planning the clinic’s daily routine, the head of the department is required to plan vacations and shifts long in advance, to extend employment contracts in good time and to establish rotation plans early on [17]. Such optimization also affects surgery planning. With few exceptions, the operation plan can be worked out at least 2 days in advance (preferably 1 week), so that residents may gain sufficient preparation time. Ideally, chief rounds should also take place at a fixed time during regular working hours – a regulation that also benefits patients and their relatives. Taking “opt-out” into account, undocumented or unpaid overtime, without the possibility of time-off in lieu, should be a thing of the past. This requires a mentality change. Weekend service is, of course, part of the surgeon’s job description, but weekend service should preferably be maximized to two per month. New work (time) models also include concepts such as job sharing, where two residents share a job. The authors conclude that not every concept is transferable to all departments. Regardless, each chief physician serves as the central role model, laying the foundation for successful structural change within the department.

## Women in surgery

In the winter semester 2017/2018, a total of 93,946 students were enrolled in human medicine in Germany and 57,765 of them were women (61.5%) [18]. Potential applicants for a training position in surgery will therefore increasingly be female. According to the *German Medical Journal*, the proportion of women in surgery is still not high compared to that of other medical fields [19]. Of 33,621 physicians working in a surgical field (including orthopedics and trauma surgery), only 5969 were female (as of December 31, 2013). That is about 18%. Surgery is generally regarded as very time consuming and therefore family-unfriendly. It must be acknowledged that household and childcare are still largely managed by women in couple relationships. This problem is reflected by a survey of 1037 members of the German Society for Gynecology and Obstetrics (DGGG) in 2010. Here, 88% of female physicians and 72% of male physicians considered career and family to be incompatible, with a correspondingly below-average number of children among gynecologists [20]. The results of the DGGG survey also showed that female gynecologists average fewer children than their male colleagues (1.06 vs. 1.68). Women in lower hierarchical positions have more children; however, in higher hierarchical positions, female gynecologists have significantly fewer children than men in the same position. This mirrors the current gender-specific situation.

The findings in the aforementioned survey are supported by a study by Bauer et al. [11] on distress in surgery. In this study, the stress prevalence of female physicians was significantly higher by 6 percentage points, at 62.4%, than that of male physicians. Conversely, male physicians were significantly more satisfied with their profession than female physicians. Of male physicians, 54.8% stated that they were very satisfied with their profession, while the percentage of satisfied female physicians was 7.4 percentage points lower. In a position paper, Roeth and Mille [17] accordingly called for a better work-life balance. These include parental leave and childcare time being taken for granted and that both parents are offered corresponding freedom by creating flexible working hours. Well-structured childcare, preferably in the form of a company kindergarten, is essential to promoting the compatibility of family and career. It should be geared to the special working conditions of hospital physicians (long opening hours to accommodate shift work, and sufficient capacity where children can also be cared for when they are sick). Flexible working hours for surgeon parents often requires collegial flexibility. It must also be made clear that individual training can take longer when such measures are implemented. Kläber [21] has accordingly stated in a report on part-time work in surgery: “In my experience, the support of the employer is not the most important aspect of success or failure of such a part-time model. The most important factor here is the acceptance of the model by the chief and, of course, by colleagues.” Under the title “A cultural change is long overdue,” the *German Medical Journal* [22] has also appealed to clinics to embed gender-balanced promotion of junior physicians into their corporate culture if they want to remain attractive employers in the future. Reference was made to the so-called “Fam-Surg” project in Lübeck [23], in which measures were developed specifically for the career development of female surgeons and for the promotion of family-friendly structures. With this model, it was shown that a balanced work-life balance is indeed feasible for surgeons, making use of part-time and shift models as well as flexible working hours.

## Conclusion

Today, the head of a surgical department is expected to have more than just core competence as a surgeon making medical decisions. Economic constraints, as well as an increasing shortage of motivated applicants for surgical training, are aspects that must be faced. Young surgeons want work schedules and models designed to ensure a work-life balance – reconciling family, leisure, career and scientific career. Satisfying these demands becomes more important the more the chief physician depends on meeting the needs of an increasing number of young women. Good working conditions include structured training. The chief physician is called upon to establish a fixed training curriculum with a clearly defined rotation plan and early and concrete planning of individual continuing education. Support for junior surgeons in their training to become surgeons, by means of mentoring programs and assistance with partial steps in complex interventions, should also be provided. To reduce work stress and prevent burnout, chief surgeons must

– reduce working requirements;
– increase scope of action by partially transferring responsibility and allowing colleagues to have a say;
– give social support through appraisal interviews, create a good working atmosphere, and prevent mobbing;
– increase rewards (possibility of further training, noticeable recognition, salary increase); and
– correct excessive collegial self-exploitation.

## Supplementary Material

The article (iss-2019-0002) offers reviewer assessments as supplementary material.
