# Supplementary material for: Surgical leadership within rapidly changing working conditions in Germany
Source: Innov Surg Sci. 2019 Apr 22;4(2):51–7. doi: 10.1515/iss-2019-0002 (PMC6754057; doi:10.1515/iss-2019-0002)
Supplement: Supplementary file 1 [file iss-4-20190002-s001.pdf]

## Reviewer Assessment

Thomas Schmitz-Rixen* and Reinhart T. Grundmann*

# Surgical leadership within rapidly changing working conditions in Germany

https://doi.org/10.1515/iss-2019-0002
Received March 4, 2019; accepted April 8, 2019

***Corresponding authors: Prof. Dr. Thomas Schmitz-Rixen,** Department of Vascular and Endovascular Surgery, Goethe-University-Hospital, Theodor-Stern-Kai 7, 60590 Frankfurt/Main, Germany, E-mail: Schmitz-Rixen@em.uni-frankfurt.de; and **Prof. Dr. Reinhart T. Grundmann,** German Institute for Vascular Public Health Research, Berlin, In den Grüben 144, 84489 Burghausen, Germany, E-mail: grundmann@medsachverstand.de

## Reviewers' Comments to Original Submission

### Reviewer 1: anonymous

Mar 09, 2019

| | |
|---|---|
| Reviewer Recommendation Term: | Accept with Minor Revision |
| Overall Reviewer Manuscript Rating: | 75 |
| Is the subject area appropriate for you? | 4 |
| Does the title clearly reflect the paper's content? | 4 |
| Does the abstract clearly reflect the paper's content? | 4 |
| Do the keywords clearly reflect the paper's content? | 4 |
| Does the introduction present the problem clearly? | 4 |
| Are the results/conclusions justified? | 4 |
| How comprehensive and up-to-date is the subject matter presented? | 3 |
| How adequate is the data presentation? | 4 |
| Are units and terminology used correctly? | 3 |
| Is the number of cases adequate? | N/A |
| Are the experimental methods/clinical studies adequate? | N/A |
| Is the length appropriate in relation to the content? | 3 |
| Does the reader get new insights from the article? | 3 |
| Please rate the practical significance. | N/A |
| Please rate the accuracy of methods. | N/A |
| Please rate the statistical evaluation and quality control. | N/A |
| Please rate the appropriateness of the figures and tables. | 3 |
| Please rate the appropriateness of the references. | 4 |
| Please evaluate the writing style and use of language. | 3 |
| Please judge the overall scientific quality of the manuscript. | 2 |
| Are you willing to review the revision of this manuscript? | Yes |

**Comments to Author:**

The authors are presenting publications on different problems with leadership in German surgery, most of them published recently. The review is interesting to read and comes to valuable conclusions. Adding a discussion, regarding the authors' position and at least some international aspects of problems in surgical leadership, would improve the quality of the manuscript.

Open Access. © 2019 Schmitz-Rixen T., Grundmann R.T., published by De Gruyter. This work is licensed under the Creative Commons Attribution 4.0 Public License.

## Reviewer 2: anonymous

Mar 21, 2019

| | |
|---|---|
| **Reviewer Recommendation Term:** | Revise with Major Modifications |
| **Overall Reviewer Manuscript Rating:** | 70 |

| | |
|---|---|
| Is the subject area appropriate for you? | 5 - High/Yes |
| Does the title clearly reflect the paper's content? | 4 |
| Does the abstract clearly reflect the paper's content? | 4 |
| Do the keywords clearly reflect the paper's content? | 3 |
| Does the introduction present the problem clearly? | 4 |
| Are the results/conclusions justified? | 4 |
| How comprehensive and up-to-date is the subject matter presented? | 5 - High/Yes |
| How adequate is the data presentation? | N/A |
| Are units and terminology used correctly? | N/A |
| Is the number of cases adequate? | N/A |
| Are the experimental methods/clinical studies adequate? | N/A |
| Is the length appropriate in relation to the content? | 4 |
| Does the reader get new insights from the article? | 4 |
| Please rate the practical significance. | 3 |
| Please rate the accuracy of methods. | N/A |
| Please rate the statistical evaluation and quality control. | N/A |
| Please rate the appropriateness of the figures and tables. | 3 |
| Please rate the appropriateness of the references. | 3 |
| Please evaluate the writing style and use of language. | 3 |
| Please judge the overall scientific quality of the manuscript. | 3 |
| Are you willing to review the revision of this manuscript? | Yes |

**Comments to Author:**
This paper gives a nice overview on the current working circumstances in surgery in Germany. However, I feel that the author should describe in more detail what exactly is meant by surgical leadership. They manuscript gives a very nice description about the working conditions but - as a reader - I wonder: what really counts to be a good surgical leader? I think it should be much more than just offering surgical Curricula for the young ones and how to get more cases. What about personality? What about communication skills? What about life-long learing for the leader? What about participation in courses like "Train the Trainer"?
I suggest that the authors add one mor section in which they clearly describe what they think is essential for a surgical leader in Germany. For the international surgical community, thia might add potential value of the manuscript.
Additionally:
1. Change title to "Surgical leadership in Germany in terms of rapidly changing working conditions"
2. The Keywords should at least have one word on leadership! The word distress should be omitted.
3. In the introduction the authors say: "..., an increasing proportion of women in medicine..." From a gender point of view, that needs to be changed! Nothing is wrong with more women in surgery! The authors could, for example, write: ...an increasing number of gender-specific challenges. The same holds true for page 10, Headline "Women in surgery" I suggest: Gender-Specific Issues in Surgery.
4. As mentioned above: I suggest one headline "Surgical Leadership - what is necessary to be a surgical leader? Here, I also recommend one more table so that the Reader gets a quicker overview.
5. During production process, the english language of the manuscript should be checked.
6. As much as possible the authors should be avoid German literature in te hreference list, because it can not be read/checked by the international readership. If it is not possible, to reduce the German references, that can be acceptes due to the fact that the paper deals with German issues.

I will be happy to review the revised manuscript again.

## Authors’ Response to Reviewer Comments

Mar 26, 2019

Dear editors,
Thank you for the review of the above-mentioned manuscript. Allow us a few comments:

- We feel that the demands of the first reviewer are already addressed in our conclusions. We can add a sentence, that the conclusions are the strong opinion of the authors.
- This paper has been written on demand, this applies to both the title and the content. It is therefore not clear to us why we should change the title.
- To the content: There is nothing more on this topic in German literature - and we will not write more about it either.
- The German situation is described only in German publications; had the international situation been given as a topic, English-language publications would inevitably have been considered.
- The unfounded criticism of the reviewer 2 irritated us when he writes: “an increasing proportion of women in medicine...” *From a gender point of view, that needs to be changed! Nothing is wrong with more women in surgery*.“ Who of us wrote this?
- Conclusion: The manuscript has already caused a lot of work. We see no reason to write an additional chapter, because there are no more data available.
- We would regret to have to withdraw the manuscript.

T. Schmitz-Rixen, R.T. Grundmann

## Editor-in-Chief Comments to Decision

Mar 30, 2019

Dear readers,
One of the major policies of „Innovative Surgical Sciences“ is a transparent and double-blind peer review process. According to the reviewers, the paper „Surgical leadership with rapidly changing working conditions in Germany” by Grundmann et al was transferred to the authors for revision. The authors answered the above-published response to the reviewer comments. In an email, the authors stated: „We do not feel able to revise the manuscript“.
Since leadership in surgery is an important issue, the Editor-in-Chief decided to publish the manuscript despite the authors‘ comments to the reviewers. „Innovative Surgical Sciences“ will be most happy to publish letters to the Editor. This may help to establish public peer-review by the readers of the article, which may also help to increase transparency in scientific publications.
